# “Vitamin D Deficiency Is More Common in Women with Autoimmune Thyroiditis: A Retrospective Study”

**DOI:** 10.1155/2021/4465563

**Published:** 2021-08-17

**Authors:** Nino Turashvili, Lali Javashvili, Elene Giorgadze

**Affiliations:** ^1^Ivane Javakhishvili Tbilisi State University, Tbilisi, Georgia; ^2^Cortex Clinic Ltd., Tbilisi, Georgia; ^3^National Institute of Endocrinology, Tbilisi, Georgia

## Abstract

**Background:**

Vitamin D is a hormone that is mainly produced in the skin upon ultraviolet B radiation exposure and has important influence on various organs. In recent years, data have been collected that vitamin D deficiency plays an important role in the development of various nonskeletal diseases, including autoimmune diseases. Chronic autoimmune thyroiditis (Hashimoto's thyroiditis) is one of the most common organ-specific autoimmune endocrine diseases. It is characterized by increased level of antithyroid peroxidase and/or antithyroglobulin antibodies in blood, which often leads to thyroid dysfunction and structural changes of the gland. There is an opinion that vitamin D deficiency may be considered as an important risk factor for development of chronic autoimmune thyroiditis, but data of various small studies are controversial. Despite the fact that Georgia is a sunny country, vitamin D deficiency is a widespread problem here. Thyroid diseases, including the chronic autoimmune thyroiditis, are also very common in Georgia. The aim of our research was to compare the level of vitamin D between the patients with chronic autoimmune thyroiditis and the healthy subjects.

**Methods:**

This retrospective study enrolled subjects, who were 18–70 years old and visited the clinics “Cortex” and “National Institute of Endocrinology” in 2018 or in 2019 from mid-spring to mid-summer. Data of thyroid-stimulating hormone, free thyroxine, antithyroid peroxidase antibodies, antithyroglobulin antibodies, thyroid ultrasonography, and 25(OH) vitamin D were retrospectively analysed based on medical history. In total, data of 1295 patients were collected. The statistical processing of data was performed through the SPSS 20 program.

**Results:**

The negative association between thyroid-stimulating hormone, antithyroid peroxidase antibodies, antithyroglobulin antibodies, heterogeneous parenchyma of thyroid gland, and vitamin D was found in women. Statistically significant association was not detected in men.

**Conclusions:**

Serum vitamin D is lower in women with autoimmune thyroiditis and primary hypothyroidism. Further studies are needed to evaluate the influence of vitamin D supplementation on thyroid autoantibody positivity or primary hypothyroidism.

## 1. Introduction

Vitamin D is a secosteroid hormone that is mainly produced in the skin upon ultraviolet B (U.V.B) radiation exposure. Other source of vitamin D is diet (in the form of D_2_ from plant sources and in the form of vitamin D_3_ from animal sources) and nutritional supplements (in the form of D_3_) or ergocalciferol (D_2_). After ingestion or synthesis, vitamin D is hydroxylated in the liver to form 25-hydroxyvitamin D (25(OH)D_2_ or 25(OH)D_3_), its major circulating form, which has little biological activity. 25(OH)D is converted in the kidney by 25(OH)D-1*α*-hydroxylase (CYP27B1) to its bioactive hormonal metabolite 1,25 dihydroxy-vitamin D (1,25(OH)_2_D or calcitriol) [1]. 1,25(OH)_2_D mediates its effects by binding to the vitamin D receptor (VDR). These receptors belong to the nuclear receptor superfamily and are expressed in nearly all tissues of the body. Therefore, vitamin D has important influence on almost all organs and cells. The concentration of serum 25(OH)D in the body is higher than serum 1,25(OH)_2_D. Therefore, the serum level of 25(OH)D is typically selected to reflect the state of vitamin D [2].

The primary action of 1,25(OH)_2_D is to enhance intestinal calcium absorption and promote osteoclast function, thereby maintaining calcium and phosphorus homeostasis and bone health [3–5]. After discovery of VDR on almost all tissues, new data have emerged providing important insights into the pleiotropic effects of vitamin D and its potential role in a variety of extraskeletal tissues, including many that affect endocrine disease (diabetes mellitus, PCOS, Addison's disease, and autoimmune thyroid disease) [6]. Moreover, recent studies indicate that 1,25(OH)_2_D is a modulator of both the innate and adaptive immune system. Immune cells such as monocytes, macrophages, dendritic cells, T-lymphocytes, and B-lymphocytes are targets for active vitamin D [7]. Active vitamin D regulates T cells: It inhibits the proliferation of T helper 1 (Th1) and decreases production of cytokines such as interleukin 2 (IL-2), tumor necrosis factor, and interferon *γ* (IFN-*γ*). Vitamin D shifts the polarization of T cells from Th1 toward Th2 that produce IL-4 and IL-5. A third group of Th cells known to be influenced by vitamin D are IL-17-secreting T cells (Th17 cells). 1,25(OH)_2_D inhibits IL-17 production. Vitamin D promotes apoptosis of dendritic cell (DC) and inhibits its differentiation and maturation. Expression of major histocompatibility complex (MHC) II on DC is downregulated by vitamin D. Tolerogenic DCs arrest the development of autoimmune diseases [8].

Hashimoto's thyroiditis (HT) is a chronic autoimmune thyroiditis, which is one of the most common organ-speciﬁc autoimmune disorder. It is characterized by increased levels of antithyroid peroxidase (anti-TPO) and/or antithyroglobulin (anti-TG) antibodies in serum, which often leads to thyroid dysfunction [9].

HT patients have a high level of Th1 cells, which produce the cytokine IFN-*γ*. Recent studies indicated that secretion of cytokines from Th17 is also involved in the development of autoimmune thyroid disease (AITD). IFN-*γ* and IFN-17A mRNA expression is significantly higher in HT patients than in healthy controls [10, 11].

According to some studies, 1,25(OH)_2_D may suppress the autoimmune process in HT at several levels, including suppression of dendritic cell-dependent T-cell activation, and decrease proliferation of Th1 cells and the synthesis of Th 1 cell cytokines such as IFN-y. Vitamin D may also inhibit the expression of MHCII surface HLA-DR antigens on thyrocytes by inhibiting the synthesis of IFN-y. In this way, vitamin D might decrease autoantibodies that react with thyroid antigens [8, 12, 13].

Recent studies have suggested a possible role of vitamin D in increasing the risk of HT development [14]; however, data regarding the possible role of vitamin D in the pathogenesis of HT are controversial: some studies suggest that vitamin D deficiency may be related to pathogenesis of HT and its supplementation could be beneficial for the treatment of HT [8, 10, 15–25]. Other studies indicate that HT is not associated with vitamin D deficiency relative to a control group [26–30].

Despite the fact that Georgia is a sunny country, vitamin D deficiency or its insufficiency is a widespread problem here. Thyroid diseases, including the chronic autoimmune thyroiditis, are also very common in Georgia.

The aim of our research was to compare the level of vitamin D between the patients with chronic autoimmune thyroiditis and the healthy subjects. The primary objective of this study was to determine the association between thyroid-stimulating hormone (TSH), free thyroxine (FT4), anti-TPO, anti-TG, and vitamin D, as well as structural changes in the thyroid gland and vitamin D. Research on this issue has never been conducted in Georgia. Research hypothesis was the following: Patients with chronic autoimmune thyroiditis may have lower level of vitamin D, compared to healthy subjects.

## 2. Methods

This retrospective study enrolled subjects, who were 18–70 years old and visited the clinics “Cortex” and “National Institute of Endocrinology” in 2018 or in 2019 from mid-spring to mid-summer. The study protocol was approved by the medical ethics committees of these clinics.

Retrospective selection of the study population was performed based on the patient's history: age 18–70 years and subjects who had lived in Georgia for a year or longer. Exclusion criteria: patients with metabolic bone diseases, primary hyperparathyroidism, hypoparathyroidism, central hypothyroidism, history of thyroidectomy, renal disorders, liver disorders, malabsorption syndromes, type 1 diabetes, obesity, granuloma-forming disorders, or epilepsy treated by anticonvulsants or patients with malignancy, immunodeficiency, and those on chronic medications that could interfere with thyroid hormone or vitamin D metabolism. Pregnant or lactating women and those who had received levothyroxine, vitamin D, or calcium supplementation in the last 6 months were also excluded.

In total, data of 1295 patients were collected. All patients had vitamin D and TSH measurement. From total 1295 participants, 866 subjects had FT4 data, 1263 had anti-TPO data, 295 had anti-TG data, and 262 had both anti-TPO and anti-TG data. Ultrasonographically, 262 subjects had the information about thyroid gland volume and echogenicity. In total, 85 subjects had thyroid nodules and information about nodule size and characteristics.

The association between study characteristics and vitamin D was statistically analysed. The laboratory and ultrasound examinations of each patient were performed within one week. The chemiluminescence immunoassay was used to analyze TSH, FT4, anti-TPO, and anti-TG levels, and the fluorescent enzyme immunoassay was used to measure vitamin D level. The thyroid and vitamin D tests have been performed on the “Tosoh AIA-900” in the same laboratory.

Serum 25(OH)D level of ≥30 ng/ml was considered as normal level and 20 to 29 ng/ml as vitamin D insufficiency, whereas serum 25(OH)D levels of <20 ng/ml were considered as an indicative of vitamin D deficiency [31]. Serum anti-TPO >34 IU/mL and/or anti-TG >115 IU/mL were considered as autoantibody positivity. Normal range for TSH was 0.27–4.2 *µ*IU/ml. Normal level of FT4 was 0.93–1.7 ng/dl (according to the local laboratory reference range). The total volume of thyroid >18 cm^3^ in female and >25 cm^3^ in male indicate thyroid enlargement [32]. Elevated TSH value was used to declare primary hypothyroidism. Elevated anti-TPO and/or anti-TG levels were used to establish the diagnosis of autoimmune thyroiditis (Hashimoto's thyroiditis).

For statistical analysis, the study population was divided according to gender and age groups for women only (<45 years and >45 y.).

The statistical processing of data was performed through the Statistical Package for the Social Sciences (SPSS) version 20 program. After the data grouping, their percentages were comparable to vitamin D subgroups (up to 20- deficiency, 20–29- insufficiency, and 30 and higher- normal), whose credibility was estimated by the *χ*^2^ test (chi-square test).

Descriptive analysis was conducted for the same groups of vitamin D, whose credibility was assessed by the ANOVA test. A *p* value of <0.05 was considered as statistically significant.

## 3. Results

The data of 1295 patients were collected, including 1097 women (84.71%) and 198 men (15.29%). The age distribution of women patients was as following: 849 (77.39%) were <45 years old and 248 (22.61%) >45 years old.

TSH and 25(OH) vitamin D was performed in all study participants. Elevated TSH level was observed in 141 subjects (10.89%), while the majority, 1154 (89.11%), had normal TSH level. Mean vitamin D value was low in both TSH level groups and corresponded to vitamin D deficiency category; the lower vitamin D level was observed in patients with primary hypothyroidism. Our study revealed a negative association between TSH and vitamin D levels in the total study population (*p*=0.008).

When we analysed the study population according to gender, we observed that 121 female subjects (11.03%) had high TSH levels. Vitamin D levels were generally low in this subgroup, and only 5.5% patients with euthyroidism and 1.7% with primary hypothyroidism had normal vitamin D status. According to our study results, a statistically significant negative association was observed between TSH and vitamin D levels in the total women population (*p*=0.01) and those whose age was <45 years (*p*=0.036). The association between TSH level and vitamin D was not statistically significant in women >45 years of age (*p*=0.232) and in male study participants (*p*=0.465).

The association between FT4 and vitamin D was not detected neither in the total study population nor in groups divided according to gender or age.

The negative association between anti-TPO and vitamin D was statistically significant in the whole study group (*p*=0.011) as well as in the group of women (*p*=0.021) and women <45 years of age (*p*=0.044).

The association between anti-TG and vitamin D was only observed in the group of women (*p*=0.022).

The association between both antibodies and vitamin D was not detected in neither group.

Thyroid ultrasonography data of 262 patients (210 females and 52 males) were analysed.

A statistically significant negative association between heterogeneous parenchyma of thyroid and vitamin D was revealed in women (*p*=0.048).

There was not also any association between total thyroid volume and vitamin D in women, as well as between the number of nodules and vitamin D and between the size of the largest nodule and vitamin D.

There was not also any association between total thyroid volume and vitamin D, heterogeneous parenchyma and vitamin D, the number of nodules and vitamin D, and between the size of largest nodule and vitamin D in the whole group as well as in men.

## 4. Discussion

Our study demonstrated the significant negative association between TSH and vitamin D, between anti-TPO and vitamin D, and anti-TG and vitamin D in women, as well as between the heterogeneous parenchyma of thyroid and vitamin D level in women.

As far as we know, this is the first research in this field in Georgia.

From total 1295 patients, 70.6% had vitamin D deficiency, 24.1% had vitamin D insufficiency, and only 5.3% had normal vitamin D level. This is an unexpected result because Georgia is a sunny country. Vitamin D deficiency was higher in hypothyroidism patients (78.7% compared to 69.6% in euthyroid people), and normal vitamin D level also was less common in hypothyroidism patients (2.8% vs. 5.6%). This result confirms our hypothesis that there is a connection between high TSH level and vitamin D deficiency.

We collected data of 1263 patients about anti-TPO and vitamin D. According to them, 71.2% had vitamin D deficiency, 23.7% had vitamin D insufficiency, and only 5.1% had normal vitamin D levels. Vitamin D deficiency was higher in patients with elevated anti-TPO (74.9% vs. 69.7%). Between 295 patients with data of anti-TG and vitamin D, 101 subjects (34.24%) had high anti-TG. Vitamin D deficiency was higher in this group, but not statistically significant.

From 866 data of FT4 and vitamin D, only 5.1% had low FT4. Vitamin D deficiency was also more common in this group (79.5 vs. 68.7%), but not statistically significant.

According to this result, we think that vitamin D deficiency is a widespread problem, but it is more common in patients in autoimmune thyroiditis and primary hypothyroidism. We think that this can be explained by vitamin D's immunomodulatory ability: Vitamin D deficiency may promote the production of various cytokines, which causes the development of autoimmune thyroiditis and often leads to decreased thyroid function.

According to gender analysis, we noted that 11.03% of women and 10.1% of men had primary hypothyroidism, but vitamin D deficiency was statistically significant only in the female group. Vitamin D was low in both groups, but the mean of vitamin D was 16.8573 in female and 18.2870 in the male group. This indicates that the link between primary hypothyroidism and vitamin D deficiency was found only in female, not in male.

Anti-TPO had been measured in 1073 female and 190 male. 30.75% in women and 13.16% in men had high anti-TPO, as well as 37.45% of 243 female and 19.23% of 52 male had high anti-TG level. A statistically significant negative association was detected only in women. This result can be explained by the fact that Hashimoto's thyroiditis is more common in women and vitamin D deficiency has been found more in women, than in men; therefore, there is a possible link between autoimmune thyroiditis and vitamin D deficiency, especially in female.

In our study, we divided the female group by age: 77.39% <45 years of age and 22.61% >45 years of age. We found a significant negative association between TSH, anti-TPO, and vitamin D only in premenopausal women. We suggest a possible link between vitamin D and estrogen in the development of AITD.

So, our retrospective study indicates that vitamin D deficiency is more common in patients (especially in women up to 45 years of age) with autoimmune thyroiditis and primary hypothyroidism than in the healthy group.

However, there are many studies with different results. For example, Yasmeh et al. studied whether vitamin D deficiency was associated with HT. Different from our study, they concluded that HT was not associated with higher rates of vitamin D deficiency relative to a control group neither in women nor in men [26]. But, in contrast to our data, in their study, the mean 25(OH)D levels for the HT and control groups were 30.75 vs. 27.56 ng/mL in females. So, none of the females were vitamin D deficient.

Similar to our research, Choi et al. described in their study that the mean of serum 25(OH)D levels was significantly lower in the TPO-Ab(+) female subjects, especially in premenopausal women. They also suggested a possible crosstalk between vitamin D and estrogen in the development of AITD [33]. Wang et al. revealed that low serum 25(OH)D is related to the presence of TG-Ab in females [19]. In their study, the prevalence of vitamin D deficiency (78.3%) and insufficiency (20%) was higher in TG-Ab-positive subjects and women had higher serum anti-TG levels and lower serum 25(OH)D_3_ than men. The authors of the survey also think about a possible crosstalk between vitamin D and X chromosome in the development of AITDs. Kim believes that serum 25(OH)D level was significantly negatively correlated with serum TSH level [18], but Musa et al. did not report any association between 25(OH) vitamin D_3_ level and hypothyroidism among females [30]. We think that the reason of this is the difference between our studies. There was a fewer number of participants (fifty-eight participants in each group of the study). Du et al. found that the incidence of thyroid nodules is lower in high level of serum 25(OH)D_3_ and serum 25(OH) D_3_ may be a protective factor for thyroid nodules [2]. However, we did not find any connection between vitamin D and thyroid nodules. The reason for this might be the high number of patients with thyroid nodules in their study. No significant association between serum 25(OH)D_3_ and the size of the largest nodule was revealed in our study. In our study, we found the negative association between heterogeneous thyroid parenchyma and low vitamin D. Heterogeneity of parenchyma is a characteristic sign for chronic autoimmune thyroiditis: lymphocytic infiltration of the thyroid leads to gradual destruction and fibrous replacement of the thyroid parenchymal tissue. In addition, Nalbant et al. showed that parenchymal blood supply of thyroid gland decreased and microvascular resistance increased in vitamin D insufficiency/deficiency. Therefore, vitamin D insufficiency/deficiency might lead to severe parenchymal injury in HT patients [34].

It should be noted that nowadays, there is a lack of consensus surrounding what constitutes optimal serum concentrations of vitamin D. Adequate serum 25-hydroxyvitamin D concentrations have been suggested to be 20 ng/ml (50 nmol/l) [35] or 30 ng/ml (75 nmol/l) [31]. However, at present, there is no agreement on “normal levels” of 25(OH)D. Several methods have been used in the quantification of vitamin D. Serum total 25(OH)D concentration remains the critical measurement for defining vitamin D status. Serum total 25(OH)D is defined as the sum of the concentrations of 25(OH)D_3_ and 25(OH)D_2_. Measurement of vitamin D status, based on currently available data, should not include the concentration of 3-epi-25(OH)D_3_ or any other vitamin D metabolites. The analytical difficulties related to the measurement of vitamin D metabolites may lead to misclassification of ‘low' vitamin D status. The variations in analytical techniques and assays may cause the large variability in laboratory measurements of vitamin D status and the systematic “errors” [36–45]. In our study, the serum 25(OH)D level of ≥30 ng/ml was considered as the normal level and 20 to 29 ng/ml as vitamin D insufficiency, whereas serum 25(OH)D levels of <20 ng/ml were considered as an indicative of vitamin D deficiency. From total 1295 patients, 70.6% had vitamin D level <20 ng/ml. So, we think that despite these different cutoff levels, the association between hypovitaminosis D and autoimmune thyroiditis would probably remain the same, even if we were to use a different level of vitamin D normality (≥20 ng/ml), but this is a subject of another study. We considered seasonal influences on vitamin D status [46], and therefore, for our retrospective study, we collected patients' data whose thyroid and vitamin D laboratory tests and thyroid ultrasound examinations were performed from mid-spring to mid-summer in 2018 or in 2019 (within one week for each patient). However, there are some limitations in our study: We could not analyse data according to patients' outdoor activity, BMI, and smoking status. Due to the analytical difficulties, in our retrospective study, we have no data about all metabolites levels of vitamin D. The number of men patients was also limited.

Thus, the results of various studies are contradictory, but for the first time, our research revealed the wide distribution of vitamin D deficiency in the population of Georgia and the possible link between the chronic autoimmune thyroiditis and primary hypothyroidism and vitamin D deficiency. However, up to now, we do not have the evidence-based data showing the efficacy of the therapeutic effect of vitamin D in improving primary hypothyroidism and decreasing thyroid autoantibody level.

## 5. Conclusions

In summary, serum vitamin D is lower in women with autoimmune thyroiditis and primary hypothyroidism. Further studies are needed to evaluate the influence of vitamin D supplementation on thyroid autoantibody positivity or primary hypothyroidism.

## Data Availability

All data generated or analysed during this study are included in this published article.

## Abbreviations

Th: T helper
IL: Interleukin
IFN: Interferon
DC: Dendritic cell
HT: Hashimoto's thyroiditis
Anti-TPO: Antithyroid peroxidase antibodies
Anti-TG: Antithyroglobulin antibodies
AITD: Autoimmune thyroid disease
TSH: Thyroid-stimulating hormone
FT4: Free thyroxine.

## Supplementary Materials

Supplementary Materials Table 1: association between TSH and vitamin D. Table 2: association between anti-TPO and vitamin D. Table 3: association between anti-TG and vitamin D in female. Table 4: association between heterogeneous parenchyma of thyroid and vitamin D in female.
