# Supplementary material for: “Vitamin D Deficiency Is More Common in Women with Autoimmune Thyroiditis: A Retrospective Study”
Source: Int J Endocrinol. 2021 Aug 17;2021:4465563. doi: 10.1155/2021/4465563 (PMC8387174; doi:10.1155/2021/4465563)
Supplement: Supplementary Materials — Table 1: association between TSH and vitamin D. Table 2: association between anti-TPO and vitamin D. Table 3: association between anti-TG and vitamin D in female. Table 4: association between heterogeneous parenchyma of thyroid and vitamin D in female. [file 4465563.f1.docx]

Table1: Association between TSH and vitamin D.

|  | | Total Number | TSH | N of Patients | Mean of vit. D | P value |
| --- | --- | --- | --- | --- | --- | --- |
| Total Patients | | 1295 | Normal TSH | 1154 | 17.2698 | 0.008 |
|  |  |  | High TSH | 141 | 15.4889 |  |
| Female | | 1097 | Normal TSH | 976 | 17.0603 | 0.01 |
|  |  |  | High TSH | 121 | 15.2198 |  |
|  | Female <45 y | 849 | Normal TSH | 768 | 17.2679 | 0.036 |
|  |  |  | High TSH | 81 | 15.4319 |  |
|  | Female >45 Y | 248 | Normal TSH | 208 | 16.2936 | 0.232 |
|  |  |  | High TSH | 40 | 14.7905 |  |
| Male | | 198 | Normal TSH | 178 | 18.4185 | 0.465 |
|  |  |  | High TSH | 20 | 17.1165 |  |

Table 2: Association between anti-TPO and vitamin D.

|  | | Total Number | Anti-TPO | N of Patients | Mean of vit. D | P value |
| --- | --- | --- | --- | --- | --- | --- |
| Total Patients | | 1263 | Normal anti-TPO | 908 | 17.3127 | 0.011 |
|  |  |  | High anti-TPO | 355 | 16.1355 |  |
| Female | | 1073 | Normal anti-TPO | 743 | 17.1429 | 0.021 |
|  |  |  | High anti-TPO | 330 | 16.0063 |  |
|  | Female <45 y | 831 | Normal anti-TPO | 592 | 17.3224 | 0.044 |
|  |  |  | High anti-TPO | 239 | 16.1721 |  |
|  | Female >45 Y | 242 | Normal anti-TPO | 151 | 16.4389 | 0.374 |
|  |  |  | High anti-TPO | 91 | 15.5710 |  |
| Male | | 190 | Normal anti-TPO | 165 | 18.0773 | 0.882 |
|  |  |  | High anti-TPO | 25 | 17.8412 |  |

Table 3: Association between anti-TG and vitamin D in female.

| Female | N | Mean of Vitamin D | P=0.022 |
| --- | --- | --- | --- |
| Normal | 152 | 20,1571 |  |
| High anti-TG | 91 | 17,7321 |  |
| Total | 243 | 19,2490 |  |

Table 4: Association between heterogeneous parenchyma of thyroid and vitamin D in female.

| Female | N | Mean of Vitamin D | P=0.048 |
| --- | --- | --- | --- |
| Homogeneous parenchyma | 36 | 19,3253 |  |
| Heterogeneous parenchyma | 174 | 16,7316 |  |
| Total | 210 | 17,1762 |  |
